# Supplementary material for: Recombinant Reg3α Prevents Islet β-Cell Apoptosis and Promotes β-Cell Regeneration
Source: Int J Mol Sci. 2022 Sep 13;23(18):10584. doi: 10.3390/ijms231810584 (PMC9504149; doi:10.3390/ijms231810584)
Supplement: Supplementary file 1 [file ijms-23-10584-s001.zip › ijms-1868625-supplementary.pdf]

SUPPLEMENTAL DATA

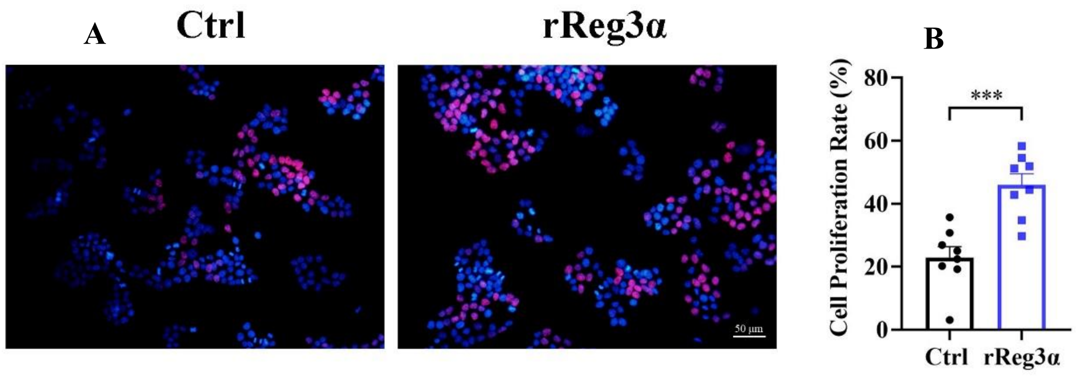

Supplementary Figure S1. rReg3α stimulates cell proliferation using EdU assay. (A) EdU staining in cells treated with rReg3α. The nuclei showing pink were labeled with EdU and considered proliferating. Scale bars: 50 μm. (B) Statistics of the proportion of proliferating cells in panel A. *** $p < 0.01$ using One-way ANOVA, $N = 8$.

WGA / DAPI

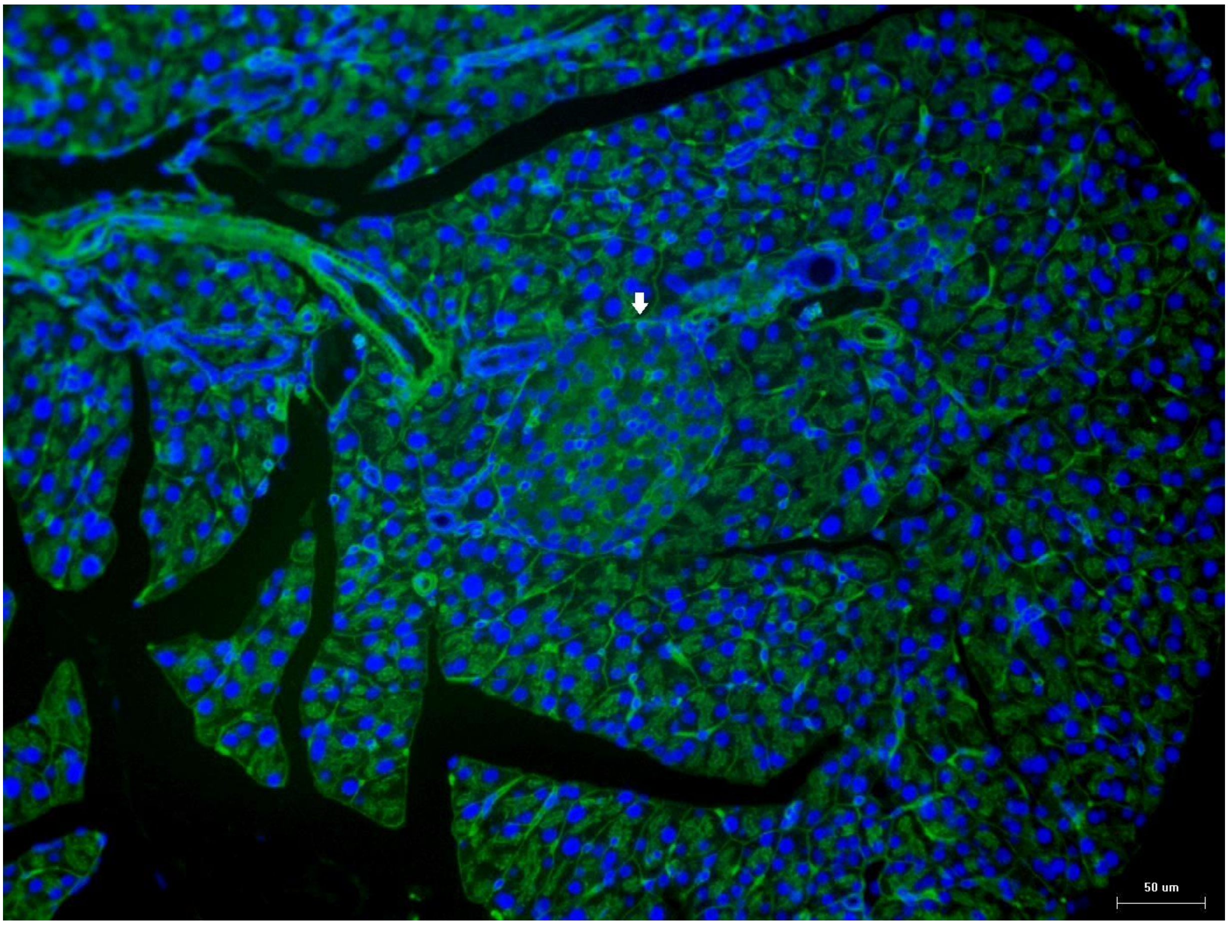

Supplementary Figure S2. WGA specifically binds to N-acetyl glucosamine on the plasma membrane of exocrine cells. In the area below the white arrow there is an endocrine islet. Although with strong background in the cytosol, WGA cannot label the plasma membrane of endocrine cells.

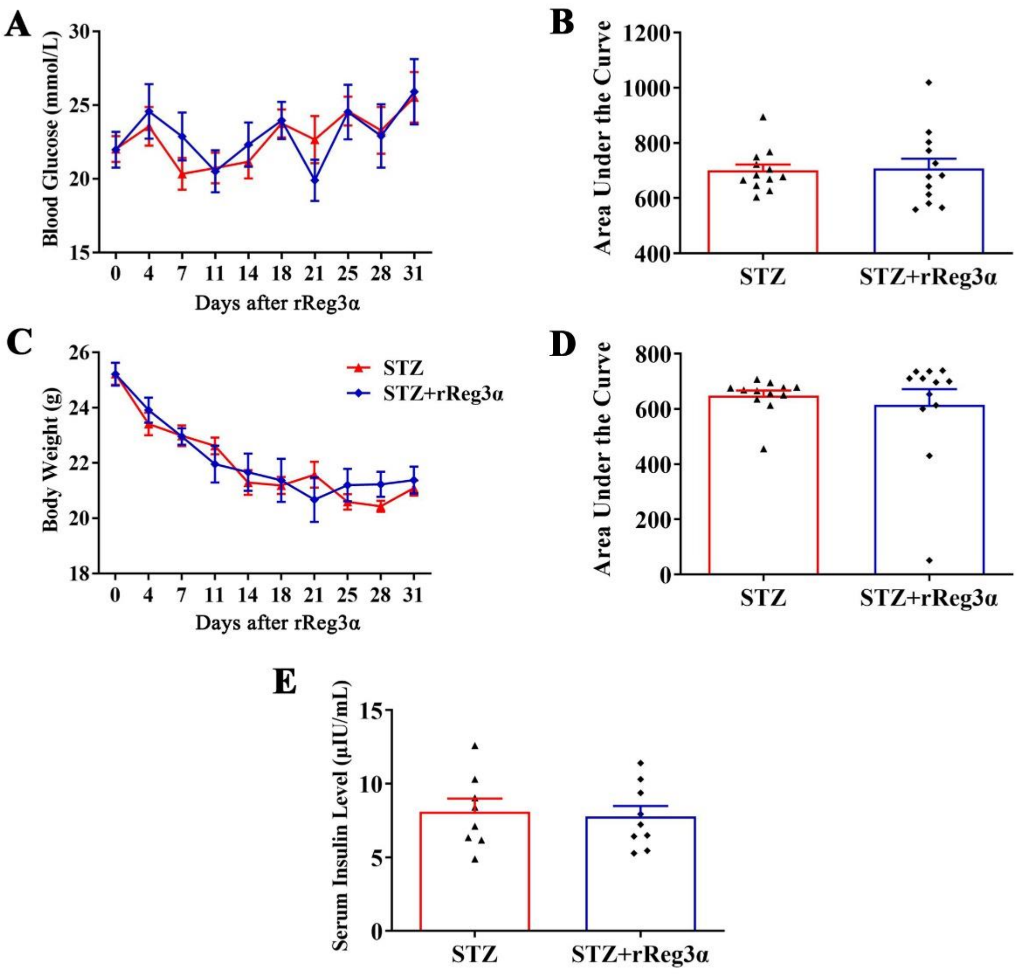

Supplementary Figure S3. rReg3α has no effect on the preexisting hyperglycemia in BALB/c mice. (A) Change in blood glucose level within 31 d after the initial rReg3α injection posterior to STZ. (B) AUC statistics of the blood glucose in panel A. (C) Change in weight loss. (D) AUC statistics of the bodyweight in panel C, $N = 12$. (E) Change in serum insulin concentration at d 31. $N = 8, 9$ (a few mice died before the endpoint). No significant change was found between groups using Student's t-test.

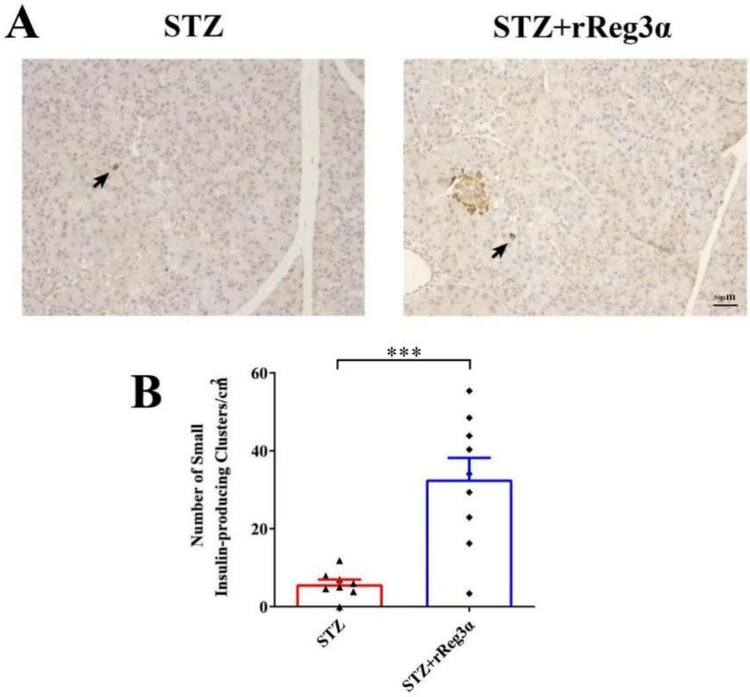

Supplementary Figure S4. rReg3α induces insulin-producing cell neogenesis in the exocrine pancreas. (A) Micrographs of immunohistochemical staining to insulin (200× magnification) at d 31 after the initial rReg3α injection posterior to STZ. A representative image was illustrated from each group. (B) Statistical analysis of the number of small insulin-producing clusters in panel A. *** $p < 0.01$ using Student's t-test, $N = 8, 9$.

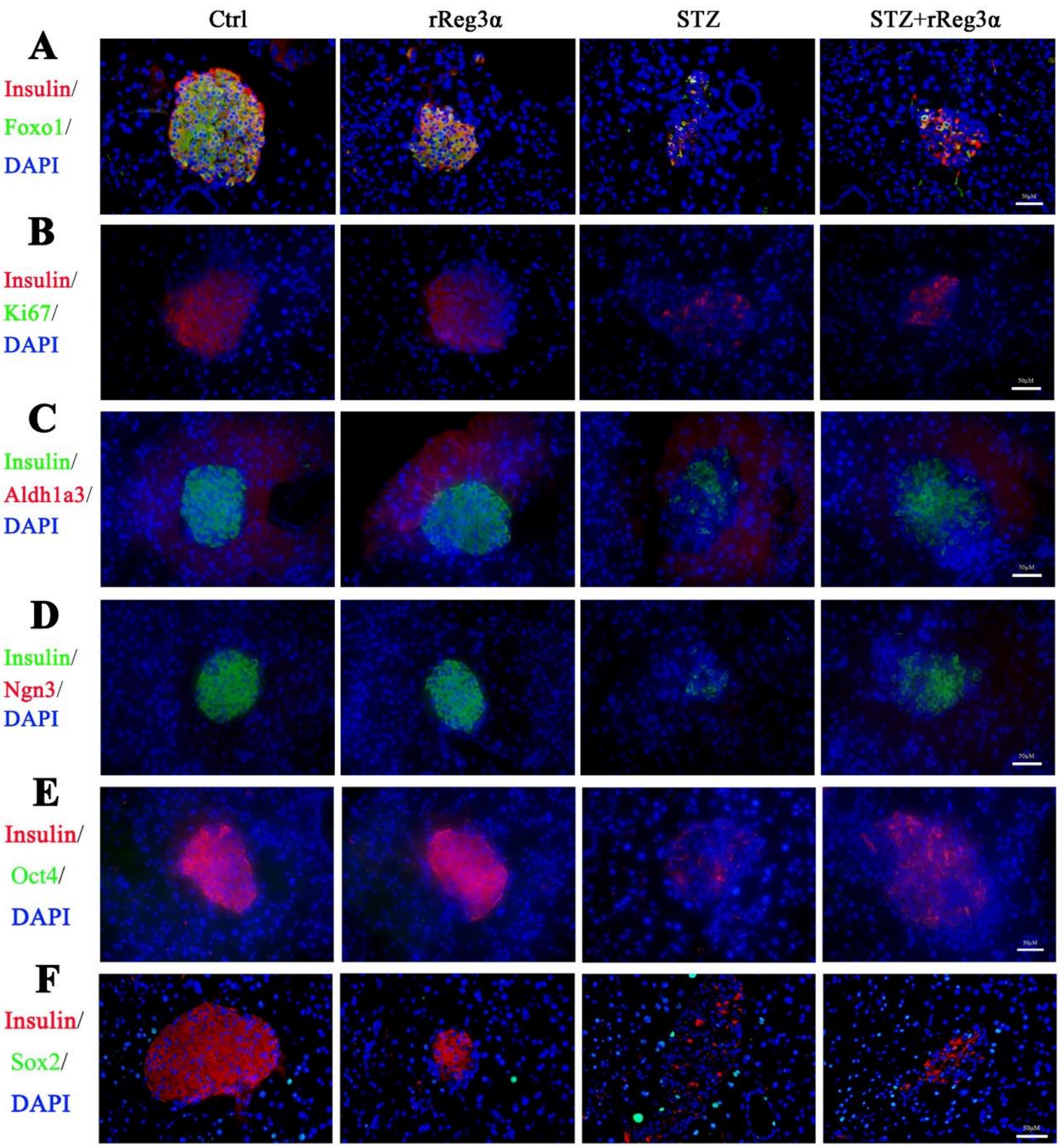

Supplementary Figure S5. Negative results of immunofluorescent staining in the pancreatic sections. (A) Foxo1 co-stained with insulin at d 2 after STZ using the same protocol of rReg3α pretreatment. No significant difference was between groups. (B) Ki67, (C) Aldh1a3, (D) Ngn3 and (E) Oct4 expression were negative. (F) Sox2 detected in a few extrainsular cells without significant difference between groups. Cell nuclei were labeled with DAPI. A representative image was illustrated from each group, $N = 5$.

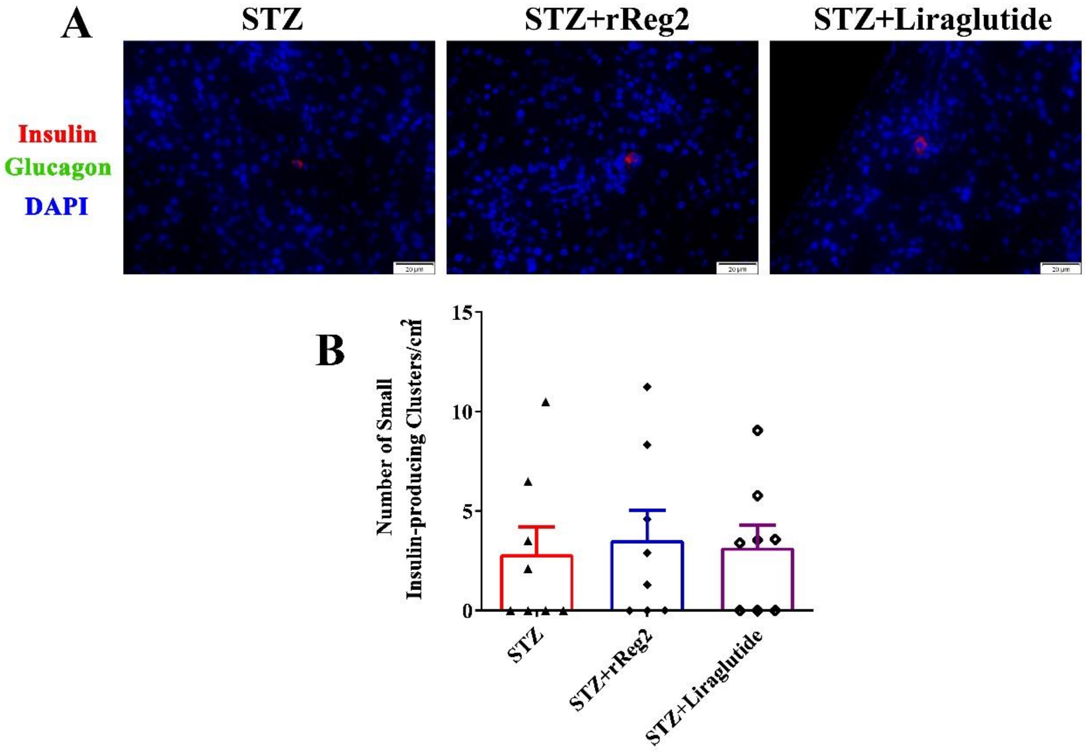

Supplementary Figure S6. Neither rReg2 nor Liraglutide increases the number of small insulin-producing cell clusters in STZ-treated BALB/c mice. (A) Using the same protocol of rReg3α pretreatment, immunofluorescent staining to insulin and glucagon (400× magnification) was performed 15 d after STZ. Cell nuclei were labeled with DAPI. A representative image was illustrated from each group. (B) Statistical analysis of the number of small insulin-producing cell clusters in panel A. No significant difference was found between groups using One-way ANOVA, $N = 8$.
